# Mental Health Innovation Is Scaling Faster Than Accountability

**DOI:** 10.7759/cureus.113012

**Published:** 2026-07-20

**Authors:** Esteban Zavaleta-Monestel, Luis Guillermo Herrera-Jiménez, Ricardo Millán González

**Affiliations:** 1 Research, Clínica Bíblica Hospital, San Jose, CRI; 2 Department of Pharmacy, University of Costa Rica, San José, CRI; 3 School of Medicine, University of Costa Rica, San José, CRI

**Keywords:** ai companions, artificial intelligence, digital mental health, mental health apps, post-market surveillance, software as medical device

## Abstract

Digital mental health tools, including chatbots, symptom checkers, artificial intelligence (AI) companions, digital therapeutics, and passive monitoring systems, are rapidly moving from peripheral wellness products into care-influencing roles. In mental health, this shift creates a distinctive accountability problem: tools may encourage disclosure, shape symptom interpretation, influence help-seeking decisions, or provide therapeutic-like interaction without assuming the duties traditionally attached to clinical care. This editorial argues that access and scalability, although important, are insufficient markers of trustworthiness. Governance should be based not only on product labels or medical-device claims, but also on the functions these systems perform and the foreseeable ways in which users rely on them. We propose a tiered model of digital mental health vigilance, with obligations that increase as tools move from low-risk wellness support toward symptom interpretation, triage-like guidance, therapeutic intervention, autonomous monitoring, or clinician-supervised treatment. Such vigilance should include clear scope limitations, privacy safeguards, user reporting channels, escalation pathways, adverse-event and near-miss reporting, performance-drift monitoring, periodic audit and explicit harm-remediation responsibilities. The analogy with pharmacovigilance is useful but incomplete because digital mental health tools evolve rapidly, lack defined exposure, and often operate without a professional intermediary. Proportionate, function-based oversight can help protect users without undermining access, particularly in low-resource settings where treatment gaps are wide and regulatory capacity may be limited. Mental health innovation may improve care, but systems that ask for clinical trust must also be prepared to account for their effects.

## Editorial

Digital mental health (DMH) has crossed a threshold. It now includes the use of digital technologies, including software, mobile applications, artificial intelligence (AI) systems, and connected monitoring tools, to support, assess, monitor, or influence mental health and mental health care. Chatbots, symptom checkers, digital therapeutics, passive monitoring applications, and AI-based companions are no longer peripheral wellness products. They are increasingly being used to interpret symptoms, invite disclosure, encourage self-management, shape decisions about seeking care, and, in some settings, extend the reach of health systems [1].

Global health bodies have warned that generative AI in health requires stronger standards for safety, transparency, and accountability [1,2]. In this editorial, "DMH tools” is used as an umbrella term, not as a homogeneous category: consumer wellness applications, AI companions, symptom checkers, regulated digital therapeutics, clinician-supervised tools, and passive monitoring systems differ substantially in function, risk, and evidentiary burden.

The question is no longer whether these tools offer promise or risk. They plainly offer both. The more important question is whether the field is prepared to build accountability as quickly as these tools are scaling. This is especially urgent in mental health, where failure is often quiet rather than spectacular. A system may offer false reassurance, provide inadequate direction toward human care, encourage dependence on simulated therapeutic attention, or normalise worsening distress without leaving a visible clinical incident. These risks are not only technical. They concern the conditions under which people decide whether to disclose distress, continue treatment, trust advice, or seek professional help.

The access argument has made DMH morally compelling. Across low- and middle-income countries (LMICs), most people with mental health conditions receive no treatment, reflecting chronic underinvestment, workforce shortages and services concentrated in urban centres [3,4]. In Latin America and the Caribbean, the treatment gap for mental disorders exceeds 77.9%. More broadly, only 2.1% of health budgets across the Region of the Americas is allocated to mental health [5]. However, access is not accountability. A product does not become trustworthy because it is scalable, widely downloaded, or easy to deploy. A tool that feels supportive may still overstate its competence. A system that increases engagement may still delay appropriate treatment.

Population-level data from two high-income countries suggest that this concern is no longer hypothetical, and there is little reason to assume that the pattern is confined to those settings. In the United States, a 2026 KFF poll found that 16% of adults had used AI tools for mental health information or advice in the preceding year, rising to 28% among adults aged 18-29 years; 7% of adults used AI to help decide whether to seek professional mental health care [6]. In a nationally representative survey, 19.2% of adolescents and young adults aged 12-21 years in the United States reported using AI chatbots for mental health advice in 2025, corresponding to more than eight million individuals; among users, 42.8% reported using them at least monthly, 91.7% rated the advice as somewhat or very helpful, and 63.3% had not disclosed this use to anyone [7]. These findings should not be read as proof that every non-consultation is harmful. They do, however, show that AI tools are already shaping care-seeking decisions outside any professional oversight, and that comparable data simply do not exist for most of the world, including the regions where the treatment gap is widest.

These usage data also expose an uncomfortable boundary problem for governance. Much of this use occurs not on purpose-built mental health products but on general-purpose conversational AI systems that make no health claims, declare no intended use and therefore sit outside any product-based regulatory perimeter. A framework that reaches only tools marketed for mental health will miss a large share of actual exposure. Function-based governance must therefore operate at two levels: product-level obligations for tools that claim or perform mental health functions, and deployment-level duties for general-purpose systems whose foreseeable use includes mental health support, such as crisis-recognition safeguards, escalation signposting, and transparent limitations. The risk-tiered architecture of the European Union’s Artificial Intelligence Act, which imposes obligations on general-purpose AI models beyond product-specific medical claims, shows that governance need not depend exclusively on declared intended use [8].

The central problem in both cases is that systems increasingly perform care-like functions while avoiding the duties normally attached to care. Some avoid explicit diagnostic or treatment claims yet provide symptom assessment, triage-like guidance, coaching, monitoring, or therapeutic conversation. They benefit from the authority and intimacy of clinical interaction while remaining positioned as consumer products. This is not to suggest that every care-like product is, or should automatically be treated as, a medical device. Formal classification depends on jurisdiction, intended use, claims, design, and applicable law. Still, for ethical and risk-based governance, function should matter at least as much as marketing language when assigning safety and accountability obligations. These concerns are especially important for adolescents and young adults, people with suicidal ideation, psychosis or paranoia, cognitive impairment, trauma histories, users in low-resource settings, and culturally or linguistically diverse populations, for whom disclosure, trust, symptom interpretation, and access to human care may be particularly fragile. A tool that influences whether users disclose severe distress, interpret symptoms in a particular way, or postpone consulting a clinician has entered ethically consequential territory, whatever its label [9].

Mental health makes this ambiguity unusually difficult to police because the boundary between support and intervention is porous. A conversational system may be marketed as a general wellbeing aid and still become, for some users, a de facto source of psychological advice, reassurance, or companionship. Evaluations of consumer-facing psychotherapy chatbots suggest that such systems may perform therapeutic functions while remaining inconsistent in transparency, safeguards, and responses to higher-risk symptoms [10]. The more a system resembles therapeutic interaction, the less plausible it becomes to treat it as a neutral consumer product.

None of this is opposition to DMH. Evidence-informed, clinically bounded and appropriately supervised tools may improve access, continuity, measurement-based care, and self-management, particularly where conventional services are thinly resourced. The problem is not digital delivery. The problem is clinical function without clinical accountability, and the corollary is that weakly governed consumer tools, regulated digital therapeutics, and clinician-integrated technologies should not be judged by the same evidentiary or operational standards.

Accountability requires more than broad commitments to responsible innovation. It requires evidence that goes beyond engagement, retention and user satisfaction: a named actor responsible for monitoring, escalation, and remediation, privacy protections suited to the intimacy of mental health disclosure, and safeguards for users who approach these systems in moments of loneliness, crisis or impaired judgement. Safety research in DMH has shown that adverse-event definitions, risk monitoring, and mitigation practices remain inconsistent, reinforcing the need for explicit safety infrastructure [11,12]. These are not peripheral implementation details. They are the minimum conditions for products that invite trust from people in distress.

Evidence standards should scale with function and risk. Tools making stronger mental health claims should provide stronger, independently evaluated evidence in the populations most likely to use them. The relevant outcomes are not downloads or time in app, but symptom change, appropriate escalation to human care, avoidance of harmful reassurance, user understanding of limitations, and performance across vulnerable groups. On November 6, 2025, the United States Food and Drug Administration (FDA)’s Digital Health Advisory Committee (DHAC) discussed generative AI-enabled DMH medical devices, including patient-facing ‘AI therapists,’ therapists, or healthcare providers, like chatbots, and other AI-based tools intended to diagnose or treat psychiatric conditions [13,14]. At that meeting, the Committee considered potential benefits and risks, risk-mitigation strategies, including alerts for self-harm ideation, human oversight, premarket evidence, postmarket monitoring, model changes and data drift, and labeling requirements. The November 6, 2025, DHAC meeting and the recommendations arising from it did not constitute binding FDA policy. FDA advisory committees provide non-binding advice and recommendations, and the FDA Executive Summary prepared for this meeting states that it was intended for discussion purposes only and did not represent draft or final guidance or propose changes to FDA policy [13,14]. Even so, it catalogues the questions regulators everywhere are now confronting: intended use, care context, human oversight, population performance, stress testing, model change, performance drift, transparent safety reporting, and patient-harm-centered risk management [13].

One of the most actionable reforms is tiered post-market surveillance for DMH tools that perform clinical or care-influencing functions. The same obligations should not apply to all tools; they should intensify as tools move from general wellbeing support toward symptom interpretation, therapeutic guidance, crisis escalation, autonomous monitoring, or clinician-supervised treatment. Crucially, tiering requires an assignment mechanism, not merely a taxonomy. A workable starting point already exists in the risk-categorisation framework for software as a medical device developed by the International Medical Device Regulators Forum, which grades obligations by the significance of the information a system provides and the criticality of the user’s health situation, and which has been endorsed across regulatory jurisdictions on several continents [15]. Tier assignment should occur at market entry based on function as designed, be revisited through change-triggered reassessment whenever models, prompts, interfaces or target populations are materially modified, and be correctable through complaint-triggered functional audit when real-world use demonstrably departs from declared scope. Without these mechanisms, function-based tiering would simply reproduce the wellness-label evasion it is meant to close.

The resulting obligations can be understood as DMH vigilance: the continuous identification, reporting, analysis, and remediation of safety, privacy, escalation, and performance failures after deployment. Low-risk wellness tools may require clear scope limitations, privacy safeguards, user reporting channels, and disclosure that the tool is not a substitute for professional care. Intermediate-risk tools providing symptom interpretation, coaching or triage-like guidance should add structured safety protocols, escalation pathways, documentation of material changes and routine review of user-reported concerns. Higher-risk tools delivering regulated treatment, autonomous monitoring, diagnostic support or clinician-supervised interventions should require independent evidence, defined accountability, adverse-event and near-miss reporting, performance-drift monitoring, periodic audit and explicit harm-remediation obligations.

The analogy with pharmacovigilance is useful but imperfect. Both medicines and DMH tools may generate harms that pre-authorisation or pre-deployment studies cannot fully anticipate. Software compounds this problem because models can be updated, interfaces redesigned, prompts altered, and user populations broadened much faster than medicines can be reformulated. However, pharmacovigilance also rests on conditions that many digital tools lack: a defined exposure, a professional intermediary who mediates access and reports suspected reactions, and established methods of causality assessment [15]. Conversational systems have no dose, no consistent professional intermediary, and harms that may be diffuse, relational, and difficult to attribute. DMH vigilance therefore needs purpose-built reporting channels, including in-product reporting designed to be usable in moments of distress, pathways for clinicians who encounter tool-related harm in practice, and analysis of aggregate system behaviour for safety-signal detection.

This need not be built from nothing. Existing World Health Organization (WHO) guidance for post-market surveillance and market surveillance of medical devices already describes the roles of regulators, manufacturers, health institutions, users, and other stakeholders in reporting, investigating, evaluating, and correcting safety problems after deployment [16]. Extending this institutional experience to care-influencing digital tools is a smaller step than inventing vigilance capacity outright. Population-level data show that AI chatbots are already being used for mental health support at a large scale; for example, a nationally representative survey estimated that more than eight million adolescents and young adults in the United States used AI chatbots for mental health advice in 2025 [7]. Treating such systems as ordinary consumer products is therefore a governance choice, not a technical necessity.

The most serious objection to this agenda deserves a direct answer: accountability has costs, and poorly designed obligations could entrench large incumbents, raise prices, and exclude low-cost tools from the very settings where the access argument is strongest. That risk is real, and it is precisely why obligations must be proportionate and tiered rather than uniform, with the heaviest duties reserved for the highest-risk functions. For countries with limited regulatory capacity, reliance and recognition mechanisms, in which national authorities draw on trusted assessments and vigilance data generated elsewhere, can deliver meaningful oversight without duplicating evaluation infrastructure, consistent with the cooperative approach promoted in the global digital health strategy [17]. Proportionality is not a loophole. It is what makes accountability compatible with access.

Privacy and data governance must be treated as core safety issues, not secondary compliance concerns. Mental health conversations, mood logs, symptom histories and crisis disclosures are among the most sensitive forms of personal information, and weak protections directly reduce candour precisely when disclosure matters most [18]. Enforcement actions against DMH companies in the United States, along with guidance on online tracking technologies, show that existing safeguards have not always matched the intimacy of the data collected [19,20].

Payment policy can turn these principles into leverage. In the United States, Medicare has begun reimbursing FDA-authorised DMH treatment devices furnished as adjuncts to clinician-supervised behavioural health care [21,22]. The structure matters more than the jurisdiction: any purchaser, whether a private insurer, a national health service or a social security system, can condition reimbursement on independent evidence, safety monitoring and transparent escalation requirements. Used well, payment policy becomes accountability policy, and it is a lever available to health systems that lack a dedicated digital health regulator [23].

These statutes are notable less for their jurisdiction than for their mechanism: they attach enforceable duties to function and foreseeable use rather than to medical claims, which is exactly the move that deployment-level governance of general-purpose AI requires [24]. The clinical stakes are not abstract. A chatbot may provide unsafe reassurance to a user describing suicidal thoughts, an AI companion may reinforce social withdrawal or emotional dependence, a symptom checker may misclassify mania, psychosis, or severe depression, passive monitoring may falsely reassure users or clinicians, and a digital therapeutic may fail to detect deterioration.

Together with WHO guidance on the ethics and governance of AI for health, including large multimodal models, and the risk-based framework of the European Union (EU) AI Act, these statutes form a repertoire of mechanisms: evidence standards, reimbursement conditions, vigilance obligations, disclosure duties, data-protection safeguards, human oversight, and post-deployment monitoring that health systems can adapt to different regulatory environments [1,8,24]. Adaptation is particularly important in low-resource settings, where the access rationale is strong, regulatory capacity may be limited, and local evidence on real-world use is often sparse [24].

Future research should move beyond adoption, engagement, and satisfaction metrics. Priorities include prospective safety studies, real-world evaluations in LMICs, audits of model drift and crisis response, studies of disclosure and dependence among adolescents and other vulnerable users, culturally and linguistically diverse validation, and comparative assessments of post-market surveillance and reimbursement conditions. Research should also measure outcomes that matter clinically, including symptom change, appropriate escalation to human care, avoidance of harmful reassurance, privacy-related harms, and user understanding of tool limitations.

Digital and AI tools may improve mental health care in meaningful ways, particularly where conventional services cannot meet demand. However, most health systems have not yet developed governance arrangements commensurate with the roles these tools are beginning to play. In mental health, trust is not a branding asset. It is part of the mechanism of care. Innovation that asks for that trust must be prepared to account for what it does with it.

## References

[1] Wallace W, Chan C, Chidambaram S (2022). The diagnostic and triage accuracy of digital and online symptom checker tools: a systematic review. NPJ Digit Med.

[2] (2025). Ethics and governance of artificial intelligence for health: Guidance on large multi-modal models. Accessed: July 7, 2026. Ethics and Governance of Artificial Intelligence for Health: Guidance on Large Multi-modal Models.

[3] (2026). World mental health report: Transforming mental health for all. Accessed. World Mental Health Report: Transforming Mental Health for All.

[4] (2018). The Lancet Commission on global mental health and sustainable development. Lancet.

[5] (2023). A New Agenda for Mental Health in the Americas: Report of the Pan American Health Organization High-Level Commission on Mental Health and COVID-19. - Google Search. Accessed: July 7, 2026. A New Agenda for Mental Health in the Americas: Report of the Pan American Health Organization High-Level Commission on Mental Health and COVID-19.

[6] (2026). KFF tracking poll on health information and trust: use of AI for health information and advice. KFF.

[7] McBain RK, Cantor JH, Breslau J (2026). AI chatbot use and disclosure for mental health among US adolescents and young adults. JAMA Pediatr.

[8] (2026). EUR-Lex: Regulation (EU) 2024/1689 of the European Parliament and of the Council of 13 June 2024 laying down harmonised rules on artificial intelligence and amending Regulations (EC) No 300/2008, (EU) No 167/2013, (EU) No 168/2013, (EU) 2018/858, (EU) 2018/1139 and (EU) 2019/2144 and Directives 2014/90/EU, (EU) 2016/797 and (EU) 2020/1828 (Artificial Intelligence Act). http://lex.europa.eu/eli/reg/2024/1689/oj/eng.

[9] Golden A, Aboujaoude E (2024). Describing the framework for AI tool assessment in mental health and applying it to a generative AI obsessive-compulsive disorder platform: tutorial. JMIR Form Res.

[10] Sobowale K, Humphrey DK, Zhao SY (2025). Evaluating generative AI psychotherapy chatbots used by youth: cross-sectional study. JMIR Ment Health.

[11] Taher R, Hsu CW, Hampshire C (2023). The safety of digital mental health interventions: systematic review and recommendations. JMIR Ment Health.

[12] Taher R, Stahl D, Shergill S, Yiend J (2025). The safety of digital mental health interventions: findings and recommendations from a qualitative study exploring users' experiences, concerns, and suggestions. JMIR Hum Factors.

[13] Tomasone JR, Flood SM, Latimer-Cheung AE (2026). US Food and Drug Administration: November 6, 2025: Digital Health Advisory Committee meeting announcement. Appl Physiol Nutr Metab.

[14] (2025). Framework to Assist Stakeholders in Technology Evaluation for Recovery (FASTER) to Mental Health and Wellness. Executive Summary for the Digital Health Advisory Committee Meeting: Generative Artificial Intelligence-Enabled Digital Mental Health Medical Devices.

[15] Sim I (2019). Mobile devices and health. N Engl J Med.

[16] (2026). Guidance for post-market surveillance and market surveillance of medical devices, including in vitro diagnostics. Accessed. Guidance for Post-market Surveillance and Market Surveillance of Medical Devices, Including in Vitro Diagnostics.

[17] (2026). Global Strategy on Digital Health 2020-2025.

[18] Huang T, Li S, Wang Y, Liu W (2026). Therapeutic interaction features of AI chatbots in depression interventions: systematic review and meta-analysis. J Med Internet Res.

[19] (2026). Federal Trade Commission: FTC gives final approval to order banning BetterHelp from sharing sensitive health data for advertising, requiring it to pay $7.8 million. Federal Trade Commission.

[20] (2026). Federal Trade Commission: Proposed FTC order will prohibit telehealth firm Cerebral from using or disclosing sensitive data for advertising purposes, and require it to pay $7 million. Federal Trade Commission.

[21] (2026). CMS.gov: Calendar year (CY) 2025 Medicare physician fee schedule final rule. https://www.cms.gov/newsroom/fact-sheets/calendar-year-cy-2025-medicare-physician-fee-schedule-final-rule.

[22] (2026). Federal Register: Medicare and Medicaid programs; CY 2026 payment policies under the physician fee schedule and other changes to Part B payment and coverage policies; Medicare shared savings program requirements; and Medicare prescription drug inflation rebate program. https://www.federalregister.gov/documents/2025/11/05/2025-19787/medicare-and-medicaid-programs-cy-2026-payment-policies-under-the-physician-fee-schedule-and-other.

[23] Ren N (2026). Current State and Challenges of Digital Health in Japan (Article in Japanese). No Shinkei Geka.

[24] Shankar R, Lim A, Xu Q (2026). Barriers and facilitators to the use of large language model-based conversational agents in mental healthcare: a systematic review. Healthcare (Basel).

